# New York Inner City Hospital COVID-19 Experience and Current Data: Retrospective Analysis at the Epicenter of the American Coronavirus Outbreak

**DOI:** 10.2196/20548

**Published:** 2020-09-18

**Authors:** Vishnu R Mani, Aleksandr Kalabin, Sebastian C Valdivieso, Max Murray-Ramcharan, Brian Donaldson

**Affiliations:** 1 Columbia University College of Physicians and Surgeons at Harlem Hospital New York, NY United States

**Keywords:** SARS-CoV-2, COVID-19, pandemic, New York City, coronavirus outbreak, American minority, outbreak, minority, mortality, patient, characteristic, mechanical ventilation

## Abstract

**Background:**

In the midst of the coronavirus disease pandemic, emerging clinical data across the world has equipped frontline health care workers, policy makers, and researchers to better understand and combat the illness.

**Objective:**

The aim of this study is to report the correlation of clinical and laboratory parameters with patients requiring mechanical ventilation and the mortality in patients infected with severe acute respiratory syndrome coronavirus 2 (SARS-CoV-2).

**Methods:**

We did a review of patients with SARS-CoV-2 confirmed infection admitted and managed by our institution during the last month. Patients were grouped into intubated and nonintubated, and subgrouped to alive and deceased. A comprehensive analysis using the following parameters were performed: age, sex, ethnicity, BMI, comorbidities, inflammatory markers, laboratory values, cardiac and renal function, electrocardiogram (EKG), chest x-ray findings, temperature, treatment groups, and hospital-acquired patients with SARS-CoV-2.

**Results:**

A total of 184 patients were included in our study with ages ranging from 28-97 years (mean 64.72 years) and including 73 females (39.67%) and 111 males (60.33%) with a mean BMI of 29.10. We had 114 African Americans (61.96%), 58 Hispanics (31.52%), 11 Asians (5.98%), and 1 Caucasian (0.54%), with a mean of 1.70 comorbidities. Overall, the mortality rate was 17.39% (n=32), 16.30% (n=30) of our patients required mechanical ventilation, and 11.41% (n=21) had hospital-acquired SARS-CoV-2 infection. Pertinent and statistically significant results were found in the intubated versus nonintubated patients with confirmed SARS-CoV-2 for the following parameters: age (*P*=.01), BMI (*P*=.07), African American ethnicity (*P*<.001), Hispanic ethnicity (*P*=.02), diabetes mellitus (*P*=.001), creatinine (*P*=.29), blood urea nitrogen (BUN; *P*=.001), procalcitonin (*P*=.03), C-reactive protein (CRP; *P*=.007), lactate dehydrogenase (LDH; *P*=.001), glucose (*P*=.01), temperature (*P*=.004), bilateral pulmonary infiltrates in chest x-rays (*P*<.001), and bilateral patchy opacity (*P*=.02). The results between the living and deceased subgroups of patients with confirmed SARS-CoV-2 (linking to or against mortality) were BMI (*P*=.04), length of stay (*P*<.001), hypertension (*P*=.02), multiple comorbidity (*P*=.045), BUN (*P*=.04), and EKG findings with arrhythmias or blocks (*P*=.02).

**Conclusions:**

We arrived at the following conclusions based on a comprehensive review of our study group, data collection, and statistical analysis. Parameters that were strongly correlated with the need for mechanical ventilation were younger age group, overweight, Hispanic ethnicity, higher core body temperature, EKG findings with sinus tachycardia, and bilateral diffuse pulmonary infiltrates on the chest x-rays. Those intubated exhibited increased disease severity with significantly elevated levels of serum procalcitonin, CRP, LDH, mean glucose, creatinine, and BUN. Mortality was strongly correlated with BMI, African American ethnicity, hypertension, presence of multiple comorbidities (with a mean of 2.32), worsening renal function with acute kidney injury or acute chronic kidney injury, and EKG findings of arrhythmias and heart blocks.

## Introduction

There is an emergent need for more research and data sharing to better delineate and understand the disease process of the coronavirus across the world. The ongoing pandemic has struck monumental changes in every aspect of human life [1]. A large amount of coronavirus cases resulted in a massive outbreak in New York State and New York City, making it the epicenter of the world [2]. Since the outbreak, rapid publications from Europe [3] and China [4] helped the rest of the world understand the disease course and severity at varying degrees. New York is one of the most culturally diverse places in the world with varying demographics across the city. Our institution in Manhattan has been at the forefront in this hotspot combating the coronavirus disease (COVID-19) pandemic at its peak. As there is a severe underrepresentation of American minority and underprivileged communities, we felt the urgent need to research and analyze various parameters associated with the disease among our patients that might help a deeper understanding and lead to further research from our peers across the world in mitigating the COVID-19 pandemic.

## Methods

### Study Design

Ethical approval from the hospital Institutional Review Board for Health Sciences Research was expeditiously sought and approved. This study entails data collection of all patients that were hospitalized and had a confirmed case of severe acute respiratory syndrome coronavirus 2 (SARS-CoV-2) infection. 

### Data Collection 

Further review using the electronic medical record was undertaken to obtain the parameters included in this study, namely, diagnosis; age; sex; ethnicity; BMI; in-hospital SARS-CoV-2 conversion status (patients without COVID-19–related symptoms upon admission and subsequently tested positive during hospital stay; hospital acquired); need for mechanical ventilation; duration of mechanical ventilation; fever (core body temperature); length of stay; death; comorbidities: hypertension (HTN), chronic kidney disease (CKD), coronary artery disease (CAD), diabetes mellitus (DM), bronchial asthma (BA), and chronic obstructive pulmonary disease (COPD); inflammatory markers: procalcitonin, C-reactive protein (CRP), lactate dehydrogenase (LDH), and ferritin; and laboratory values: creatinine (Cr), blood urea nitrogen (BUN), D-dimers, prothrombin time (PT), partial thromboplastin time, troponin, blood natriuretic peptide (BNP), glucose, electrocardiogram (EKG), chest x-ray (CXR) findings, and treatment received (azithromycin, hydroxychloroquine). 

### Inclusion and Exclusion Criteria

Out of 204 total patients, 184 were admitted with high suspicion of coronavirus infection or other diagnoses (who later had a conversion) between March and April 2020 and were included in our study. The included patients were confirmed positive for SARS-CoV-2. Of the 204 patients, 20 were excluded, as they tested negative. 

### Statistical Analysis 

Baseline characteristics of all patients with laboratory confirmed SARS-CoV-2 were included in our study and described in detail. Gross numbers with percentages were calculated and used. We summarized the continuous variables using mean and standard deviation, whereas categorical variables were summarized using counts and percentages. Continuous variables were primarily analyzed using the student *t* test with Welch correction, while categorical variable analysis were accomplished using the Fisher exact test; subgroup analyses were performed using a two-tailed chi-square test. *P*<.05 was considered statistically significant. A Microsoft Excel (Microsoft Corporation) spreadsheet was used to display extrapolated data. Graph Pad Prism 8 (Graph Pad Software) and R software version 4.0.0 (R Project for Statistical Computing) were used for statistical analysis.

## Results

### Definitions

Patients included in our study for the purposes of understanding the disease severity and statistical analysis were further classified into the following groups: the entire group (termed total), nonintubated total (NI-T), nonintubated living (NI-L), nonintubated deceased (NI-D), intubated total (I-T), intubated living (I-L), and intubated deceased (I-D). We compared parameters across all patients and groups to delineate deeper clinical understanding with associations as detailed further in the results and Multimedia Appendix 1. 

### Patient Characteristics 

The total number of patients included in our study was 184 of the total 204 as detailed in the inclusion and exclusion criteria in the Methods section. All of our patients were SARS-CoV-2 confirmed by polymerase chain reaction of the collected nasopharyngeal sample. 

The overall mortality in our study was 17.39% (n=32). In our study, 16.30% (n=30) required mechanical ventilation, and the mortality rate was 43.33% (n=13/30) among those intubated. Of the 184 patients, 21 (11.41%) had contracted SARS-CoV-2 in the hospital who were otherwise admitted for other reasons, mostly surgical. The average age was 64.72 (range 28-97, SD 14.87) years. Over one-third of the patients included were female and less than two-thirds were male. Mean age with SD for all groups is reported in Table 1. We found that younger patients were more likely to be intubated than their older counterparts. The comparison between NI-T and I-T yielded a *P* value of .01, and the comparison between NI-L and NI-D yielded a *P* value of .51, and between I-L and I-D yielded a *P* value of .60. 

**Table 1:** Baseline patient characteristics and descriptive statistics.

Patient characteristics		Total (N=184)	NI-T^a^ (n=154)	I-T^b^ (n=30)	*P* value	NI-L^c^ (n=135)	NI-D^d^ (n=19)	*P* value	I-L^e^ (n=17)	I-D^f^ (n=13)	*P* value
Age (years), mean (SD)		64.72 (14.87)	65.75 (15.25)	59.47 (11.56)	.01	64.58 (15.04)	74.05 (14.52)	.51	58.47 (12.19)	60.77 (11.03)	.59
**Sex, n (%)**					.04			.45			.10
	Females	73 (39.67)	61 (39.61)	12 (40)		55 (40.74)	6 (31.58)		9 (52.94)	3 (23.08)	
	Males	111 (60.33)	93 (60.39)	18 (60)		80 (59.26)	13 (68.42)		8 (47.06)	10 (76.92)	
**BMI (kg/m^2^), mean (SD)**		29.10 (7.386)	28.45 (7.017)	31.74 (8.645)	.07	29.00 (7.066)	25.71 (6.116)	.04	33.98 (10.07)	28.57 (4.913)	.07
	<24.9, n (%)	50 (29.24)	45 (31.69)	5 (17.24)		35 (28.46)	10 (52.63)		3 (17.65)	2 (16.67)	
	25.0-29.9 (overweight), n (%)	55 (32.16)	46 (32.39)	9 (31.03)		41 (33.33)	5 (26.32)		2 (1.76)	7 (58.33)	
	30.0-34.9 (class 1 obesity), n (%)	39 (22.80)	31 (21.83)	8 (27.59)		29 (23.58)	2 (10.53)		7 (41.18)	1 (8.33)	
	35.0-39.9 (class 2 obesity), n (%)	16 (9.36)	11 (7.75)	5 (17.24)		9 (7.32)	2 (10.53)		3 (17.65)	2 (16.67)	
	>40.0 (class 3 obesity), n (%)	11 (6.43)	9 (6.34)	2 (6.90)		9 (7.32)	0 (0)		2 (11.76)	0 (0)	
**Ethnicity, n (%)**											
	African American	114 (61.96)	101 (65.58)	13 (43.33)	<.001	86 (63.70)	15 (78.95)	.19	10 (58.82)	3 (23.07)	.05
	Hispanic	58 (31.52)	43 (27.92)	15 (50)	.02	39 (28.89)	4 (21.05)	.48	6 (35.30)	9 (69.23)	.07
	Asian	11 (5.98)	9 (5.84)	2 (6.67)	N/A^g^	9 (6.67)	0 (0.00)	N/A	1 (5.88)	1 (7.7)	N/A
	Caucasians	1 (0.54)	1 (0.65)	0 (0.00)	N/A	1 (0.74)	0 (0.00)	N/A	0 (0.00)	0 (0.00)	N/A
Length of stay (days), mean (SD)		15.15 (13.45)	14.85 (14.42)	16.67 (6.434)	.28	15.76 (15.12)	8.37 (3.833)	<.001	20.88 (4.833)	11.15 (3.288)	<.001
Temperature (°F), mean (SD)		100.9 (1.680)	100.7 (1.568)	101.8 (1.928)	.004	100.7 (1.551)	100.4 (1.694)	.37	102.1 (1.703)	101.5 (2.225)	.49

^a^NI-T: nonintubated total.

^b^I-T: intubated total.

^c^NI-L: nonintubated living.

^d^NI-D: nonintubated deceased.

^e^I-L: intubated living.

^f^I-D: intubated deceased.

^g^Not applicable.

### BMI

Of the 184 patients, the mean for BMI was 29.10 (SD 7.386, range 13.38-61.68) kg/m^2^, of which more than one-fourth had a BMI<24.0 kg/m^2^, less than one-tenth had a BMI of 25-29.9 kg/m^2^, less than one-fourth were in class 1, and class 2 and class 3 each had less than one-tenth. We then analyzed to see if the BMI was associated with either mortality or the increased need for mechanical ventilation. We found that BMI had correlations for both NI-T and I-T, with means of 28.56 and 31.74, respectively (*P*=.07); NI-L and NI-D had means of 29 and 25.71, respectively (*P*=.04), and I-L and I-D had means of 33.98 and 28.57, respectively (*P*=.07).

### Ethnicity

Of the 184 patients, less than one-third were Hispanic, more than three-fifths were African Americans, less than one-tenth were Asians, and there was 1 Caucasian. For African Americans, the comparison of NI-T and I-T had a *P* value <.001; I-L and I-D yielded a *P* value of .05. For Hispanics, a comparison of NI-T and I-T yielded a *P* value of .02; I-L and I-D had a *P* value of .07. All subgroup analysis findings are listed in Table 1. 

### Comorbidities

The average comorbidity was 1.70 for all patients admitted with SARS-CoV-2 infection. The average comorbidity among patients in each group were NI-T: 1.73; I-T: 1.57; NI-L: 1.64; NI-D: 2.32; I-L: 1.41; and I-D: 1.77. There was statistical significance with a *P* value of .045 on comparing NI-L and NI-D, clearly indicating that increased comorbidity among patients not requiring mechanical ventilation positively correlated with mortality. We then calculated percent associations for each group based on the presence of existing comorbidities. Overall, almost two-thirds of the overall group had HTN. There was statistical significance when comparing NI-L and NI-D (*P*=.02), indicative of a positive correlation of HTN with mortality; there was no difference between the I-T and NI-T (*P*=0.91). Overall, less than one-fifth of the patients had CKD; there was no statistical significance when comparing all 3 groups for CKD as depicted in Table 2. Overall, about one-fifth had CAD. There were no statistical differences noted among the groups for CAD; however, a comparison of NI-L and NI-D yielded a *P*=.07. Overall, over two-fifths of our patient population had DM. We had statistical significance between the NI-T and I-T for DM (*P*<.001). Both BA and COPD made up less than one-tenth of our patients. There was no significance among any groups for BA and COPD. Table 2 is representative of the percent associations of various comorbidities with *P* values for the groups compared.

**Table 2:** Association of comorbidities in patients with severe acute respiratory syndrome coronavirus 2.

Comorbidity	Total (N=184), n (%)	NI-T^a^ (n=154), n (%)	I-T^b^ (n=30), n (%)	*P* value	NI-L^c^ (n=135), n (%)	NI-D^d^ (n=19), n (%)	*P* value	I-L^e^ (n=17), n (%)	I-D^f^ (n=13), n (%)	*P* value
Hypertension	12 (65.76)	101 (65.68)	20 (66.67)	.91	84 (62.22)	17 (89.47)	.02	10 (58.82)	10 (76.92)	.30
Chronic kidney disease	32 (17.39)	28 (18.18)	4 (13.33)	.52	22 (16.30)	6 (31.58)	.11	1 (5.89)	3 (23.08)	.17
Coronary artery disease	37 (20.11)	32 (20.78)	5 (16.67)	.61	25 (18.52)	7 (36.84)	.07	2 (11.76)	3 (23.08)	.41
Diabetes mellitus	80 (43.48)	75 (48.70)	5 (16.67)	.001	66 (48.89)	9 (47.37)	.90	2 (11.76)	3 (23.08)	.41
Bronchial asthma	18 (9.78)	17 (11.04)	1 (3.33)	.19	13 (9.63)	4 (21.05)	.14	0 (0.00)	1 (7.69)	.24
Chronic obstructive pulmonary disease	17 (9.24)	16 (10.39)	1 (3.33)	.22	13 (9.63)	3 (15.79)	.41	0 (0.00)	1 (7.69)	.24

^a^NI-T: nonintubated total.

^b^I-T: intubated total.

^c^NI-L: nonintubated living.

^d^NI-D: nonintubated deceased.

^e^I-L: intubated living.

^f^I-D: intubated deceased.

### Inflammatory Markers

We then assessed the distribution of inflammatory markers across various subgroups to gain an understanding of them and to see if this would serve as an indirect surrogate marker of the disease severity. Details of inflammatory markers along with other laboratory values with range, mean, SD, and the standard error of the mean (SEM) for each subgroup are depicted in Table 3 along with the results of the statistical analysis.

**Table 3:** Inflammatory markers and laboratory values in patients with confirmed severe acute respiratory syndrome coronavirus 2.

Parameters		Total		I-T^a^		NI-T^b^			*P* value			NI-L^c^			NI-D^d^			*P* value			I-L^e^			I-D^f^			*P* value	
**Creatinine (mg/dL)**								.03									.10									.77		
	Mean (SD)		2.738 (3.177)		4.030 (3.481)		2.486 (3.063)						2.286 (2.879)			3.908 (3.953)						4.194 (3.811)			3.815 (3.134)			
	Range		0.40-15.80		0.60-12.30		0.40-15.80						0.40-15.60			0.70-15.80						0.60-12.30			0.70-10.90			
	SEM^g^		0.2342		0.6355		0.24688						0.2477			0.9068						0.9244			0.8692			
	95% CI		2.276-3.200		2.730-5.330		1.999-2.974						1.796-2.776			2.003-5.814						2.235-6.154			1.922-5.71			
**Blood urea nitrogen (mg/dL)**								.001									.04									.56		
	Mean (SD)		45.30 (38.07)		68.47 (40.69)		40.79 (35.97)						37.76 (33.21)			62.26 (47.27)						64.53 (38.14)			73.62 (44.84)			
	Range		6-234		10-169		6-234						6-234			19-188						22-134			10-169			
	SEM		2.807		7.429		2.899						2.858			10.84						9.251			12.44			
	95% CI		39.76-50.84		53.27-83.66		35.06-46.51						32.11-43.42			39.48-85.05						44.92-84.14			46.52-100.7			
**Procalcitonin (ng/dL)**								.03									.20									.23		
	Mean (SD)		2.463 (7.091)		7.809 (13.62)		1.435 (4.348)						1.237 (4.209)			3.380 (5.363)						4.581 (7.071)			12.65 (19.33)			
	Range		0.03-56.42		0.13-56.42		0.03-43.95						0.03-43.95			0.14-16.24						0.13-24.44			0.15-56.42			
	SEM		0.5696		2.724		0.3814						0.3875			1.548						1.826			6.112			
	95% CI		1.338-3.588		2.186-13.43		0.681-2.190						0.47-2.005			–0.028 to 6.79						0.665-8.497			–1.18 to 26.48			
**C-reactive protein (mg/L)**								.01									.24									.76		
	Mean (SD)		17.99 (14.28)		24.06 (11.60)		15.39 (14.61)						13.47			34.97						24.70			23.16			
	Range		0.1-91.80		3.3-44.82		0.1-91.80						0.1-34.46			3.64-91.80						3.3-44.82			3.34-35.57			
	SEM		1.596		2.368		1.953						1.385			15.41						3.172			3.726			
	95% CI		14.81-21.17		19.16-28.96		11.48-19.30						10.69-16.25			–7.81 to 77.76						17.85-31.56			14.73-31.58			
**D-dimer (ng/ml)**								.45									.47									.17		
	Mean (SD)		5200 (11,363)		6636 (10,959)		4512 (11,598)						3733 (10,070)			13463 (23,656)						8565 (13,643)			3420 (976.5)			
	Range		177-56,475		694-53,952		177-56,475						177-56,475			839-48,935						694-53,952			1236-4857			
	SEM		1321		2237		1640						1885			11,828						3523			325.5			
	95% CI		2568-7833		2008-11,263		1215-7808						742.6-6724			–24,180 to 51105						1010-16,120			2670-4171			
**Lactate dehydrogenase (U/L)**								.001									.57									.34		
	Mean (SD)		572.8 (233.4)		723 (234.2)		519.6 (210.3)						523.8 (215.5)			480.3 (163.1)						678.3 (227.3)			776.6 (242.9)			
	Range		165-1345		387-1345		165-1009						165-1009			279-671						387-1069			567-1345			
	SEM		25.47		49.94		26.71						28.80			66.57						65.62			76.83			
	95% CI		522.2-623.5		619.1-826.9		466.2-573						466-581.5			309.2-651.5						533.9-822.8			602.8-950.4			
**Ferritin (ng/ml)**								.95									.24									.52		
	Mean (SD)		1678 (2678)		1702 (1932)		1667 (2952)						1717 (3086)			1148 (489.6)						1897 (2405)			1429 (1021)			
	Range		36-17,860		84-9366		36-17,860						36-17,860			739-1901						264-9366			84-2899			
	SEM		297.6		394.4		391						427.9			218.9						642.9			322.8			
	95% CI		1085-2217		885.4-2518		884.1-2451						858.2-2576			540.2-1756						507.8-3285			698.7-2159			
**Prothrombin time (sec)**								.49									.65									.57		
	Mean (SD)		14.38 (3.46)		14.87 (2.889)		14.24 (3.612)						14.06 (2.740)			16.58 (10.09)						15.16 (3.208)			14.30 (2.330)			
	Range		10.20-31.17		10.50-20.80		10.20-31.17						10.20-25.50			11.10-31.70						10.50-20.80			11.80-17			
	SEM		0.4135		0.7459		0.4870						0.3837			5.046						1.015			1.042			
	95% CI		13.55-15.20		13.27-16.47		13.27-15.22						13.29-14.83			0.5163-32.63						12.86-17.46			11.41-17.19			
**Partial thromboplastin time (sec)**								.16									.67									.84		
	Mean (SD)		39.29 (21.97)		50.86 (37.09)		35.77 (13.47)						35.94 (13.96)			34.05 (7.175)						52.39 (39.49)			48.10 (36.57)			
	Range		19.80-120		22.90-120		19.80-108.7						19.80-108.7			27.60-42.80						22.90-120			23.50-109			
	SEM		2.837		9.913		1.986						2.155			3.587						13.16			16.35			
	95% CI		33.62-44.97		29.44-72.27		31.77-39.77						31.59-40.29			22.63-45.47						22.03-82.75			2.696-93.50			
**Troponin (ng/ml)**								.72									.29									.49		
	Mean (SD)		0.04039 (0.09629)		0.04862 (0.1185)		0.03853 (0.09118)						0.03198 (0.07648)			0.09290 (0.1674)						0.02964 (0.03198)			0.06950 (0.1708)			
	Range		0.01-0.56		0.01-0.5550		0.01-0.56						0.01-0.4920			0.01-0.56						0.01-0.0970			0.01-0.5550			
	SEM		0.009018		0.02587		0.009455						0.008395			0.05292						0.009643			0.05401			
	95% CI		0.023-0.058		–0.005 to 0.103		0.02-0.057						0.016-0.049			–0.027 to 0.213						0.008-0.051			–0.053 to 0.19			
**Brain natriuretic peptide (pg/ml)**								.12									.56									N/A^h^		
	Mean (SD)		4061 (12,513)		1260 (1223)		4419 (13,254)						4596 (13,975)			2927 (3931)						1295 (1364)			1085 (0)			
	Range		14-70,000		23-3379		14-70,000						14-70,000			164-9815						23-3379			1085			
	SEM		1719		499.1		1933						2156			1758						609.8			0			
	95% CI		612.1-7510		–23.40 to 2543		527-8310						241.5-8951			–1955 to 7808						–398.5 to 2988			0			
**Glucose (mg/ml)**								.01									.18									.07		
	Mean (SD)		231.3 (169.8)		297.3 (145.7)		218.4 (171.5)						218.4 (171.5)			287.2 (208.2)						253.9 (124.2)			354.2 (156.8)			
	Range		78-1466		135-720		78-1466						78-1466			119-899						135-576			157-720			
	SEM		12.51		26.61		13.82						13.82			47.76						30.12			43.49			
	95% CI		206.6-256		242.9-351.8		191.1-145.7						191.1-145.7			186.8-387.5						190-317.7			259.4-448.9			

^a^I-T: intubated total.

^b^NI-T: nonintubated total.

^c^NI-L: nonintubated living.

^d^NI-D: nonintubated deceased.

^e^I-L: intubated living.

^f^I-D: intubated deceased.

^g^SEM: standard error of the mean.

^h^Not applicable.

### Procalcitonin

There was significant statistical difference between the NI-T and I-T groups (95% CI 0.7077-12.04, *P*=.03); however, we found no difference between NI-D and NI-L (95% CI 1.322-5.607, *P*=.20) or between I-D and I-L (*P*=.23).

### C-Reactive Protein

Comparison of NI-T and I-T yielded a *P* value of .006 (95% CI 2.516-14.82), and a comparison of NI-D and NI-L had a *P* value of .24 (95% CI –21.20 to 64.19). Finally, I-D compared to I-L had a *P* value of .76 (95% CI –11.77 to 8.871).

### Ferritin

There was no statistical significance in all 3 comparison groups, which were as follows: NI-T and I-T (95% CI 1075-1144, *P*=.95), NI-D and NI-L (95% CI –1538 to 400, *P*=.24), I-D and I-L (95% CI –1975 to 1040, *P*=.52).

### Lactate Dehydrogenase

We found that there was statistical significance of LDH between NI-T and I-T (95% CI 88.31-318.6, *P*=.001), while no differences were found between I-D and I-L or the NI-D and NI-L groups (*P*=.57 and *P*=.34, respectively). 

### Fever

The mean temperature was 100.9° F (SD 1.68, SEM 0.1239) across all patients. Plotted values for each subgroup are shown in Table 1. On comparing the temperature between NI-T and I-T, we found statistical significance (*P*=.004) with means of 100.7° F and 101.8° F, respectively. There was no significant difference linking temperature to mortality among the remainder of the subgroup analysis.

### Prothrombin Time

There was no statistical significance across all three groups for PT: NI-T vs I-T (95% CI –1.1997 to 2.457, *P*=.49), NI-D vs NI-L (95% CI –13.49 to 18.52, *P*=.65), and I-D vs I-L (*P*=.57).

### Partial Thromboplastin Time

No statistical difference between all 3 comparison groups with the following: *P*=.16, *P*=.67, and *P*=.84 comparing NI-T vs I-T and then between the NI and I subgroups, respectively. 

### D-Dimers

D-dimers were elevated on most of our patients with SARS-CoV-2 confirmed cases. There were no significant differences among the groups; although, the intubated patients had a mean that showed a clear trend of elevation compared to those nonintubated. NI-T vs I-T had a *P* value of .45, with a mean for NI-T of 4512 and for I-T of 6636. Further analysis between the deceased and living patients of the nonintubated and intubated groups yielded *P*=.47 and *P*=.17, respectively. 

### Glucose

There was statistical significance between the NI-T and I-T groups, who had mean glucose levels of 218.4 and 297.3, respectively (95% CI 18.46-139.3, *P*=.01). There was no significance between the nonintubated subgroups NI-D and NI-L (*P*=.18); however, it is noteworthy that the mean glucose levels among the deceased were 287.2 compared to 218.4 for the living. We noted the same trends among the intubated group with a mean of 354.2 among the deceased vs 253.9 for I-L. The comparison of I-D vs I-L yielded a *P* value of .07. 

### Creatinine

There was a clear trend of increasing Cr values, indicating acute kidney injury (AKI) proportional to the disease severity regardless of their pre-existing renal function status. NI-T vs I-T was statistically significant (*P*=.03), with a mean Cr of 2.486 and 4.030, respectively (95% CI 0.1641-2.924). Although there was no statistical significance among the NI-D vs NI-L, it is quite clear that up trending Cr was indicative of worsening renal function among the deceased *P*=.09. The patients intubated overall had worse renal function, but there was no significance between groups (*P*=.77). 

### Blood Urea Nitrogen

We performed the same grouped analysis for BUN. There was significant differences correlating that of Cr among the NI-T and I-T (95% CI 11.54-43.82, *P*=.001). There was statistical significance among the living and deceased in the nonintubated population (95% CI 1.148-47.85, *P*=.04). No statistical differences were detected between the I-L and I-D group (*P*=.56).

### Troponin

Although troponin had been elevated, indicating myocardial stress, in most of our patients that had it drawn, a correction was done for those with and without pre-existing CAD and potential influence with coexisting CKD. We did note an elevation across all groups. NI-T and I-T had means of 0.03853 and 0.04862, respectively (*P*=.72); NI-D vs NI-L had a *P* value of .28, and I-D vs I-L had a *P* value of .48. 

### Brain Natriuretic Peptide

There was no significance for all three groups. Of note, we had lower sample size and reduced power in the groups after correction; only a few of our patients had BNP drawn. Among those that had it drawn, most of them had their values elevated. NI-T vs I-T had a *P* value of .12, and NI-D vs NI-L had a *P* value of .56.

### Electrocardiogram

Our EKG findings among patients and their distribution across various subgroups are described in Multimedia Appendix 1. Predominant EKG findings were sinus rhythm (n=94/160, 58.75%); sinus tachycardia (n=34, 21.25%); sinus tachycardia with prolonged QT (n=3, 1.875%); prolonged QT (n=6, 3.75%); and arrhythmia, blocks, or other (n=23, 14.38%). Comparison of NI-T and I-T yielded a *P* value of .08 for tachycardia; comparison of NI-L and NI-D had a *P* value of .02 for arrhythmias and heart blocks, indicating that patients with pre-existing arrhythmias or heart blocks had positive associations with mortality. 

### Chest X-Ray

We classified CXR findings into 7 major classes as reported by the radiologist for all the patients in the study group. We sorted out associations as depicted in Table 4 with percent distributions, group analysis, and *P* values. Of significance, we found that NI-T and I-T comparisons were statistically significant (*P*=.02) for bilateral (B/L) patchy opacities, which was a predominant finding among the nonintubated patients and with B/L diffuse pulmonary infiltrates predominantly in the intubated group, for which the comparison of NIT to I-T was statistically significant (*P*<.001). 

**Table 4:** Chest x-ray associations with the coronavirus disease.

CXR^a^ findings	Total (N=184), n (%)	Nonintubated total (n=154), n (%)	Intubated total (n=30), n (%)	*P* value	Nonintubated living (n=135), n (%)	Nonintubated deceased (n=19), n (%)	*P* value	Intubated living (n=17), n (%)	Intubated deceased (n=13), n (%)	*P* value
Patchy bilateral opacity	51 (27.72)	48 (31.17)	3 (10)	.02	42 (31.11)	6 (31.58)	.97	1 (5.88)	2 (15.38)	.40
Patchy unilateral opacity	23 (12.5)	21 (13.64)	2 (6.67)	.30	19 (14.08)	2 (10.53)	.67	1 (5.89)	1 (7.69)	.84
Diffuse bilateral infiltrates	84 (45.65)	60 (38.96)	24 (80)	<.001	51 (37.78)	9 (47.37)	.42	14 (82.35)	10 (76.92)	.71
Diffuse unilateral infiltrate	1 (0.54)	1 (0.65)	0 (0)	.66	1 (0.74)	0 (0)	.71	0 (0)	0 (0)	N/A^b^
Ground glass opacity	2 (1.09)	2 (1.30)	0 (0)	.53	2 (1.48)	0 (0)	.59	0 (0)	0 (0)	N/A
Increased interstitial marking	13 (7.07)	12 (7.79)	1 (3.33)	.38	10 (7.41)	2 (10.53)	.64	1 (5.88)	0 (0)	.37
Normal CXR	10 (5.43)	10 (6.49)	0 (0)	.15	10 (7.41)	0 (0)	.22	0 (0)	0 (0)	N/A

^a^CXR: chest x-ray.

^b^Not applicable.

### Treatment

Of the total 184 patients, 153 patients received some form of treatment as categorized in Table 5 of our manuscript. The primary treatment groups were group 1 receiving azithromycin and hydroxychloroquine, group 2 receiving Azithromycin only, group 3 receiving hydroxychloroquine only, and group 4 no treatment. They were not grouped based on any specific parameters, but treatments were given with emerging science and potential alleviation of the disease process. Percent distribution and *P* values are described in Table 5. We found that no combination of treatment made any difference in preventing the need for mechanical ventilation or mortality. 

**Table 5:** Treatment groups for the coronavirus disease.

Treatment	Total (N=184), n (%)	Nonintubated (n=154), n (%)	Nonintubated living (n=135), n (%)	Nonintubated deceased (n=19), n (%)	Intubated (n=30), n (%)	Intubated living (n=17), n (%)	Intubated deceased (n=13), n (%)
Azithromycin and hydroxychloroquine	90 (48.91)	71 (46.10)	63 (46.67)	8 (42.11)	19 (63.33)	11 (64.71)	8 (61.54)
Azithromycin	23 (12.5)	23 (14.94)	20 (14.81)	3 (15.79)	0 (0)	0 (0)	0 (0)
Hydroxychloroquine	40 (21.74)	31 (20.13)	27 (20)	4 (21.05)	9 (30)	5 (29.41)	4 (30.77)
No treatment	31 (16.85)	29 (18.83)	25 (18.52)	4 (21.05)	2 (6.67)	1 (5.88)	1 (7.69)

## Discussion

Our study population was primarily grouped into patients that required admission with and without the need for mechanical ventilation, we further classified and subgrouped them to deceased and living, this renders a deeper perspective on the entire spectrum of COVID-19 among the hospitalized. Our patient population is unique in that it gives a fair representation of the American minority. It was interesting to note that in our study younger patients were more likely to need mechanical ventilation than their older counterparts, while the older patients had increased mortality, and this positively correlated with increasing comorbidity. We also found that the comparatively lower BMI group had worse prognosis in both needing mechanical ventilation and mortality contrary to our traditional beliefs [5]. Increased core body temperature corresponded to worsening disease. Our study had striking differences in that more African Americans with increased comorbidities had higher mortality and decreased need for mechanical ventilation compared to Hispanics, who were more likely to be intubated and then succumb to the disease. HTN was independently associated with mortality among the nonintubated. Inflammatory markers were increased across the board for all of our patients with significant differences among the I-T and NI-T groups and trends between the deceased and living; this certainly proves that they act as surrogate markers of disease severity in SARS-CoV-2 infection. Recent studies linked AKI to COVID-19 [6], our study not only confirmed their findings but also showed directly proportional worsening of renal function with increasing disease severity. Mean blood glucose followed the same trajectory, corresponding to disease severity indicative of insulin resistance insurgence [7]. Pre-existing conduction defects, atrial fibrillation, and arrhythmias positively correlated with mortality among the nonintubated. We also noted that patients with B/L CXR findings of patchy opacities were more likely to be hospitalized without the need for mechanical ventilation and recovery versus those with B/L diffuse infiltrates, who were more likely to be intubated. Our study suggests azithromycin and hydroxychloroquine treatment when given individually or as combination therapy did not alter disease progression and mortality.

Although our study clearly illustrates associations of age, BMI, ethnicity, body temperature, multiple comorbidities, HTN, DM, CAD, inflammatory markers, mean blood glucose, AKI, EKG findings, and CXR findings with likelihood of needing intubation or mortality, it is not without limitations. This is an early retrospective and not a randomized controlled study. Sample size, demographics, and ethnic composite in our study needs to be taken into account while interpreting results. We urge the readers, clinicians, and researchers to take our early findings, bearing in mind its limitations while making clinical decisions. Further investigation and research on a large scale based on our early findings is essential. 

## Appendix

Multimedia Appendix 1 Electrocardiogram findings.

## Abbreviations

AKI: acute kidney injury
BA: bronchial asthma
BNP: blood natriuretic peptide
BUN: blood urea nitrogen
B/L: bilateral
CAD: coronary artery disease
CKD: chronic kidney disease
COPD: chronic obstructive pulmonary disease
COVID-19: coronavirus disease
Cr: creatinine
CRP: C-reactive protein
CXR: chest x-ray
DM: diabetes mellitus
EKG: electrocardiogram
HTN: hypertension
I-D: intubated deceased
I-L: intubated living
I-T: intubated total
LDH: lactate dehydrogenase
NI-D: nonintubated deceased
NI-L: nonintubated living
NI-T: nonintubated total
PT: prothrombin time
SARS-CoV-2: severe acute respiratory syndrome coronavirus 2
SEM: standard error of the mean
