# Supplementary material for: New York Inner City Hospital COVID-19 Experience and Current Data: Retrospective Analysis at the Epicenter of the American Coronavirus Outbreak
Source: J Med Internet Res. 2020 Sep 18;22(9):e20548. doi: 10.2196/20548 (PMC7505684; doi:10.2196/20548)
Supplement: Multimedia Appendix 1 [file jmir_v22i9e20548_app1.docx]

EKG findings

| **Groups**  **EKG** | **Total**  **(T)**  **N = 160(184)** | **Non-Intubated**  **Total**  **(NI-T)**  **N =137(154)** | **Non-Intubated Living**  **(NI-L)**  **N =122(135)** | **Non-Intubated Deceased (NI-D)**  **N = 15(19)** | **Intubated**  **(I-T)**  **N = 23(30)** | **Intubated Living**  **(I-L)**  **N = 13(17)** | **Intubated Deceased**  **(I-D)**  **N = 10(13)** |
| --- | --- | --- | --- | --- | --- | --- | --- |
| **Normal Sinus Rhythm** | 94 (58.75%) | 84 (61.31%) | 77 (63.11%) | 7 (46.67%) | 10 (43.48%) | 6 (46.15%) | 4 (40%) |
| **Sinus Tachycardia** | 34 (21.25%) | 26 (18.98%) | 24 (19.67%) | 2 (13.33%) | 8 (34.78%) | 5 (38.46%) | 3 (30%) |
| **Sinus Tachycardia + Prolonged QT** | 3 (1.875%) | 2 (1.46%) | 2 (1.64%) | 0 (0%) | 1 (4.35%) | 1 (7.70%) | 0 (0%) |
| **Prolonged QT** | 6 (3.75%) | 6 (4.38%) | 5 (4.10%) | 1 (6.67%) | 0 (0%) | 0 (0%) | 0 (0%) |
| **Arrhythmia / Blocks / Others** | 23 (14.38%) | 19 (13.87%) | 14 (11.48%) | 5 (33.33%) | 4 (17.40%) | 1 (7.70%) | 3 (30%) |
| **No EKG** | 24 (13.04%) | 17 (11.03%) | 13 (9.63%) | 4 (21.05%) | 7 (23.33%) | 4 (23.56%) | 3 (23.08%) |
